# Supplementary material for: Large area inkjet-printed OLED fabrication with solution-processed TADF ink
Source: Nat Commun. 2023 Nov 9;14:7220. doi: 10.1038/s41467-023-43014-7 (PMC10632475; doi:10.1038/s41467-023-43014-7)
Supplement: Supplementary file 1 — Supplementary Information [file 41467_2023_43014_MOESM1_ESM.pdf]

Supplementary Information

Large area inkjet-printed OLED fabrication with solution-processed TADF ink

Chandra Kant[1,2], Atul Shukla[3,4], Sarah K. M. McGregor[3,5], Shih-Chun Lo[3,5,], Ebinazar B. Namdas[3,4,*], Monica Katiyar[1,2,*]*

[1] *Materials Science and Engineering Department, Indian Institute of Technology Kanpur, India*

[2] *National Centre for Flexible Electronics, Indian Institute of Technology Kanpur, India*
[3] *Centre for Organic Photonics & Electronics, The University of Queensland, Brisbane Australia*

[4] *School of Mathematics and Physics, The University of Queensland, Brisbane, Australia*
[5] *School of Chemistry and Molecular Biosciences, The University of Queensland, Brisbane, Australia*

*Corresponding author: mk@iitk.ac.in, e.namdas@uq.edu.au & s.lo@uq.edu.au

Supplementary Note 1.

Reagents and starting materials were purchased from commercial suppliers and used without further purification. Anhydrous toluene was dried over 4Å molecular sieves. Merck aluminium plates coated with 60 F254 silica gel were used for thin layer chromatography (TLC) and visualised using 254 nm (short wave) and 365 nm (long wave) light. Column chromatography was carried out using Merck LC60A 40-30 silica gel. Solvent mixtures are reported by volume. ^{1}H NMR was recorded using Bruker Avance 300 MHz spectrometer in $CDCl_3$, and chemical shifts (δ) were reported in parts per million (ppm) and referenced to the residual solvent peak, i.e., δ 7.26 ppm ($CHCl_3$ for ^{1}H). Multiplicities were reported as singlet (s), doublet (d), doublet of doublets (dd), doublet of doublets of doublets (ddd), and multiple (m). All coupling constants (*J*) were quoted in Hertz (Hz) and rounded to the nearest 0.5 Hz. LRMS was collected using Thermo LCQ Fleet Ion Trap in ESI positive mode and analysed using the on-board Xcalibur software. Peaks recorded as a mass to charge ratio (*m/z*).

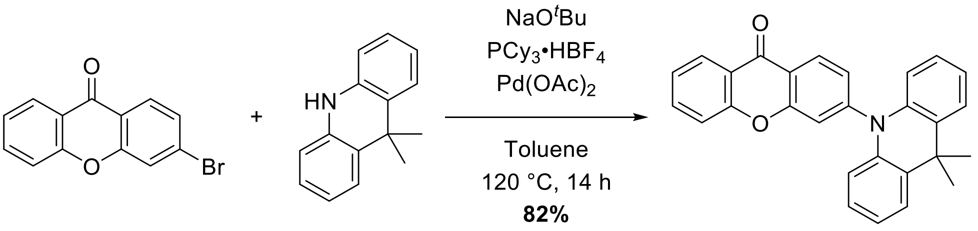

ACRXTN

Supplementary Figure S1. Synthesis scheme of TADF material, ACRXTN. TADF material, 3-(9,9-dimethylacridin-10(9*H*)-yl)-9*H*-xanthen-9-one (ACRXTN), was synthesized with modification to a reported procedure.[1] To an oven dried Schlenk flask under argon, 9,9-dimethyl-9,10-dihydroacridine (86.0 mg, 0.411 mmol), 3-bromo-9*H*-xanthen-9-one (101 mg, 0.367 mmol), anhydrous toluene (5 mL), sodium *tert*-butoxide (85.0 mg, 0.884 mmol), tricyclohexylphosphine tetrafluoroborate (13.5 mg, 0.037 mmol), and palladium acetate (20.0 mg, 0.089 mmol) were added. While stirring, the mixture was deoxygenated by carefully evacuating and backfilling with argon three times. The flask was wrapped with aluminium foil to exclude light, and heated at reflux in an oil bath at 120 °C for 14 hours under argon. The mixture was allowed to cool to room temperature before being filtered through celite and washed with additional toluene (50 mL). The filtrate was collected, and solvent was removed under reduced pressure. The crude product was purified over silica chromatography using dichloromethane as eluent, and then further purified by recrystallisation from ethyl

acetate/methanol (1:2) to give ACRXTN as a yellow crystalline solid (120 mg, 81%). The characterisation data are in accordance with those reported[1].

TLC: (dichloromethane, R_f): 0.74 (UV). ^{1}H NMR (300 MHz, $CDCl_3$): δ 1.68 (s, 6H), 6.49–6.57 (m, 2H), 6.98–7.08 (m, 4H), 7.38 (dd, J = 8.5 & 2.0 Hz, 1H), 7.40–7.46 (m, 1H), 7.46–7.56 (m, 4H), 7.72–7.79 (m, 1H), 8.38 (ddd, J = 8.0 & 2.0 & 0.5 Hz, 1H), 8.53 (dd, J = 8.5 Hz & 0.5 Hz, 1H). LRMS: m/z calculated for $C_{28}H_{22}NO_2$ [M+H]: 404.16 (100%), 405.17 (30%), 406.17 (4%), found: 404.36 (100%), 405.36 (34%) 406.32 (6%).

^{1}H NMR spectra of ACRTXN

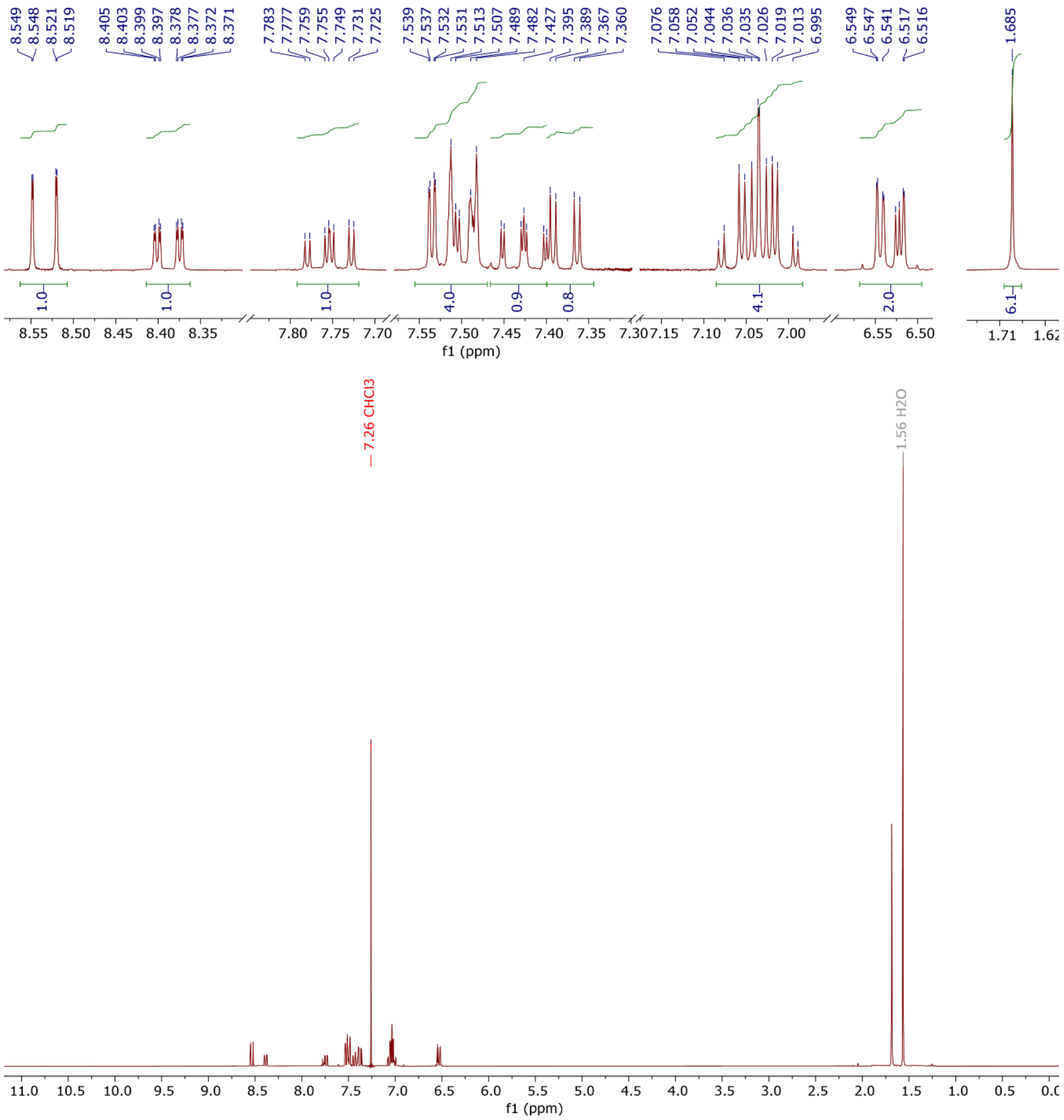

Supplementary Figure S2. ^{1}H NMR (300 MHz, $CDCl_3$) of ACRXTN with the expansion shown on the top.

Supplementary Note 2.

We utilized commonly used organic solvents such as 1,2-dichloroethane (DCE), chlorobenzene (CB), 1,2-dichlorobenzene (*o*-DCB), toluene, and methyl benzoate (MB). The physical properties of these solvents can be found in Table S1. First, DCE was selected as the starting solvent for ink formulation. After stirring (800 RPM) and heating the ink at 40 ^{o}C for two hours, we obtained an excellent translucent ink. However, within half an hour at room temperature the ink began to settle and become cloudy, indicating an unsuitable formulation for printing (Figure S2a- Ink 1). Replacing DCE with CB, the same conditions were then used to formulate a second ink, which was encouragingly found to have extended solution stability at room temperature (Figure S2a Ink 2). Using this formulation, we could create excellent and durable ink. However, the CB evaporated rapidly through the nozzles while not printing, leaving the residues of the solid material at the nozzle plate and resulting in the nozzle clogging after just a few minutes of repeated jetting cycles (Figure S2b). This led to poor droplet directivity and significantly decreased film formation and reproducibility.

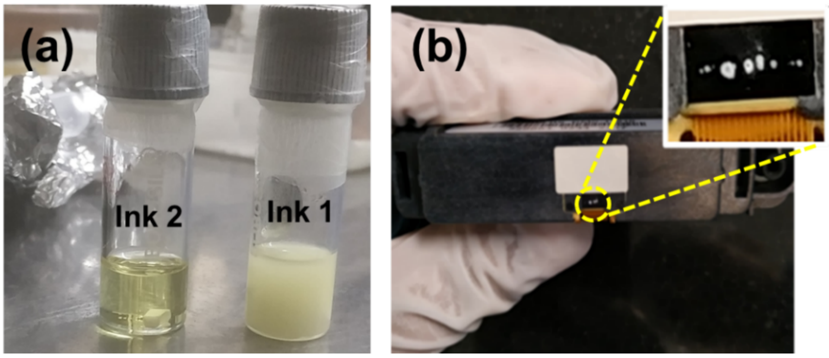

Supplementary Figure S3. Images of ink solutions and nozzle clogging. a Ink 1 with TADF/CBP in DCE and Ink 2 with TADF/CBP in CB solvent after one-hour. **b** Ink 2 shows nozzle clogging after a few jetting cycles of printing.

To overcome the nozzle clogging issue, we turned our focus to use *o*-DCB as it has a low vapour pressure and a higher boiling point, while maintaining comparable structural and solvation properties to CB. However, over a short period of time (15 mins), we observed that the directivity of the droplets for single solvent ink formulation (*o*-DCB) changed as the same applied pulse was changed. This might be because chlorinated solvent (*o*-DCB) etched the nozzle plate. Although ink formulation and jetting with a single higher boiling point solvent is possible, the long-term stability of the ink solution may be a concern in achieving uniform films. Therefore, we explored binary solvent systems with complimentary boiling

temperatures, viscosities, and surface tensions. The printability of the finished solution is heavily impacted by the solvent's chemical and physical characteristics. The boiling point of a solvent and its surface tension are often correlated. The balance of these two factors has a considerable impact on the droplet formation that is produced overall and modifies the pace at which solvents evaporate at the contact between the nozzle and air.

Supplementary Note 3.

The Ohnesorge (Oh) number is inversely proportional to the Z number. It is a dimensionless constant and is used to explain the propensity of a drop to either remain together or disintegrate. The Oh number is connected to the Reynolds number (Re), which can be written as $Re = V \rho l \eta^{-1}$, and the Weber number (We), which can be written as $We = V^2 \rho l \sigma^{-1}$, where V is the droplet velocity, ρ is the mixture density, l is the characteristic length and equal to the nozzle diameter (D, 21 μm in this work), and σ is the surface tension. This tendency is determined by comparing the forces exerted by viscous fluid with the forces exerted by inertial motion and surface tension. Equation 1 below provides the expression for Oh,

$$Oh = \eta/(\rho\sigma D)^{0.5} \quad (1)$$

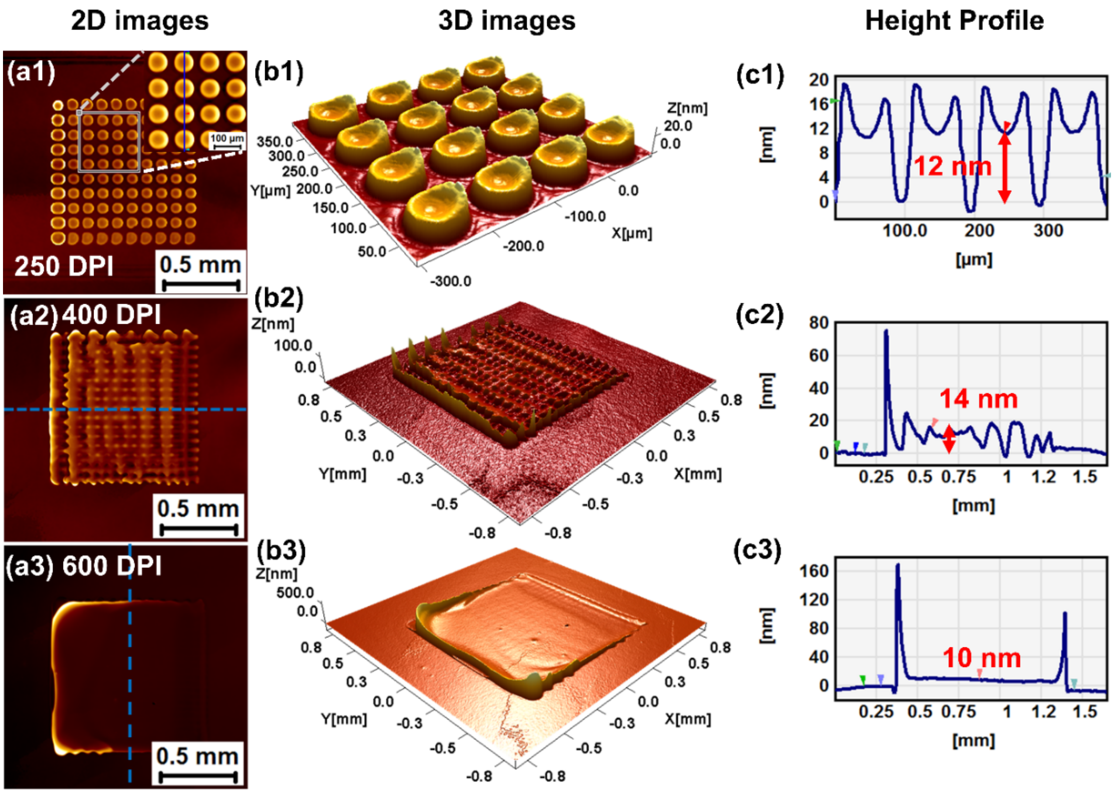

Supplementary Figure S4. Inkjet printing of 5.5 mg mL^{-1} ink on PVK:TAPC/PEDOT:PSS/ITO. a1-3 2D-image of printed patterns of 1 × 1 mm^2 with 250, 400 and 600 DPI. b1-3 3D images of printed droplets and square patterns with 250, 400 and 600 DPI. c1-3 Height-profile of printed single droplets from 250, 400 and 600 DPI. With an ink concentration of 5.5 mg mL^{-1}, we found that, on average, the height was 10–12 nm from the centre point of printed droplets. However, the height at the edges were quite high. This is because at lower concentrations, solid contents were deposited more heavily in the periphery due to capillary flow and the fact that evaporation begins at the edges[4,5]. At 600 DPI, fully covered films started to form with an average height of 10 ±2 nm. However, the sharp edges (160 nm) formed at the periphery were many times thicker than the average film thickness. This is problematic since such edges will cause shorts between layers in the devices. Hence, we used higher concentrations of ink formulation.

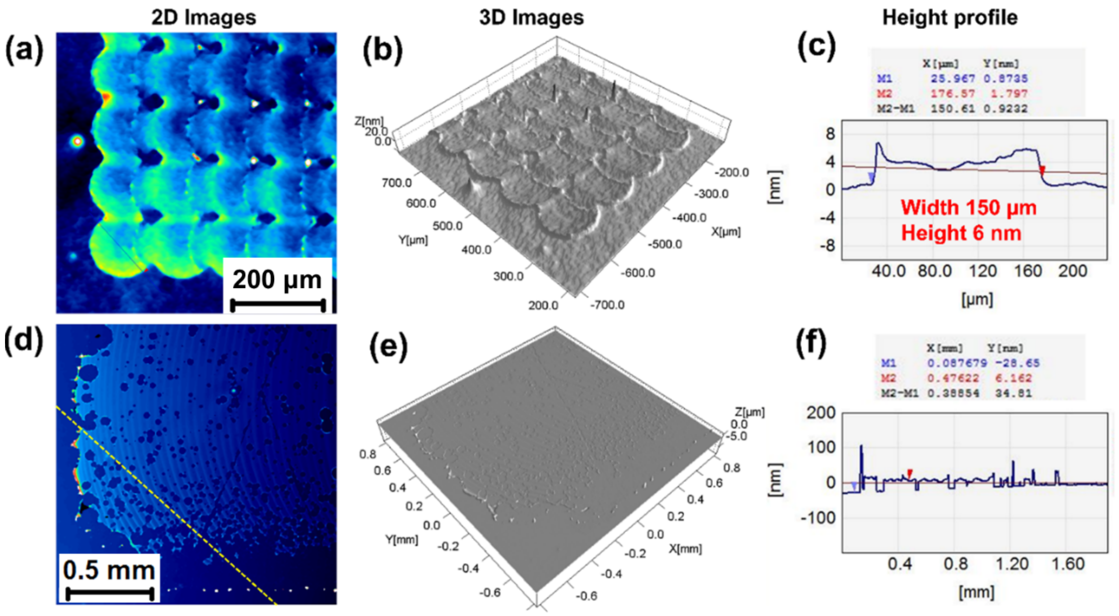

Supplementary Figure S5. Ink jet printing of TADF ink on PEDOT:PSS films. **a** 2D-image of the printed droplets with 200 DPI. **b** 3D images of printed droplets with 200 DPI. **c** Height-profile of single droplet printed with 200 DPI having drop diameter of 150 ± 4 µm and height of 6 ± 2 nm. **d** 2D image of the printed droplets at 600 DPI but after drying the film showing non-uniform as many patches can be seen. **e** 3D profile of the same film. **f** Cross-sectional height profile showing the discontinuous film when printed at 600 DPI.

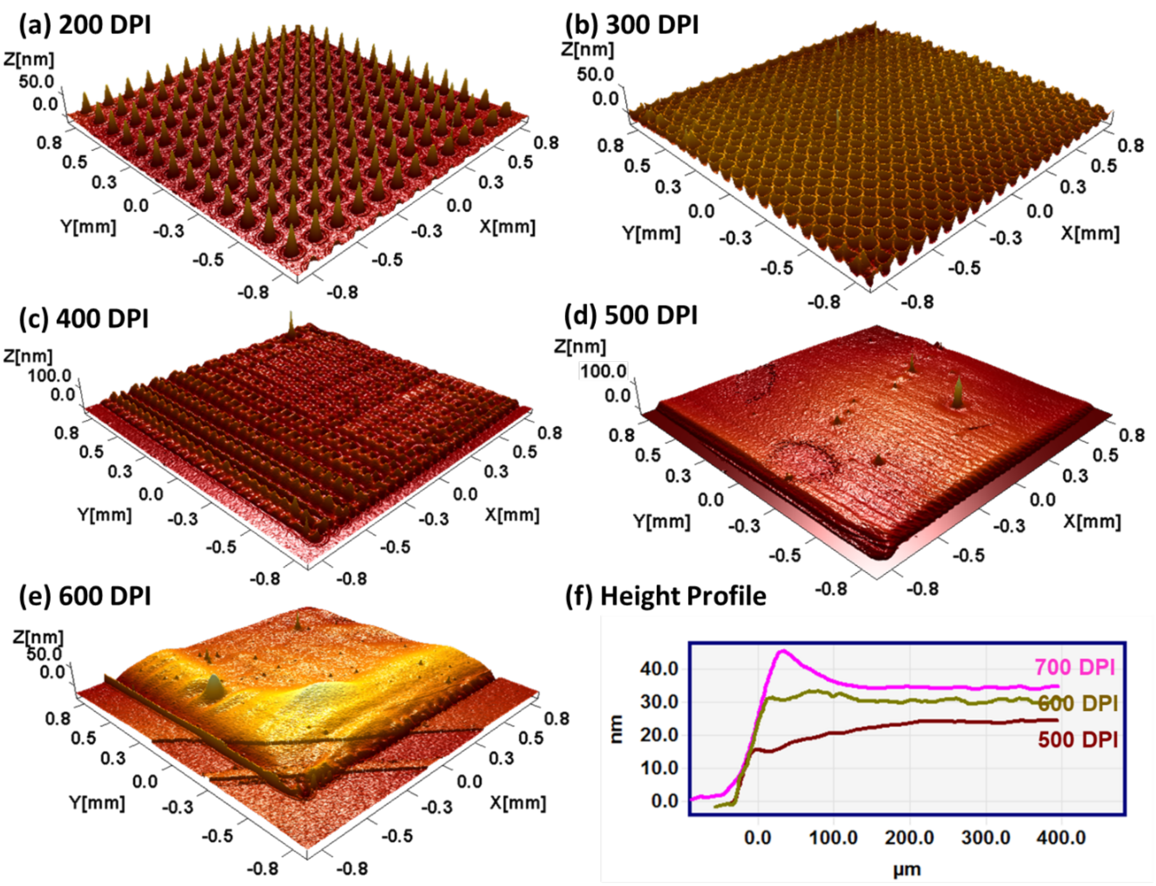

Supplementary Figure S6. 3D-images of printed droplets patterns (11.25 mg L^{-1} TADF ink). The 3D images of printed droplets patterns with different DPIs at 40 °C substrate temperature, a Printed with 200 DPI, b 300 DPI, c 400 DPI, d 500 DPI, e and 600 DPI. f Cross sectional height profiles.

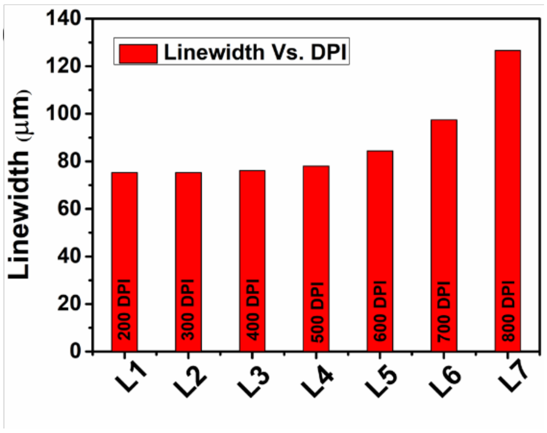

Supplementary Figure S7. The linewidth broadening of single IJP lines. L1 to L7 with varying DPI (200–800) as DPI increases from 200 to 800 (L1 to L7) is 75.25, 75.25, 76.10, 77.99, 84.41, 97.45, and 126.62 μm.

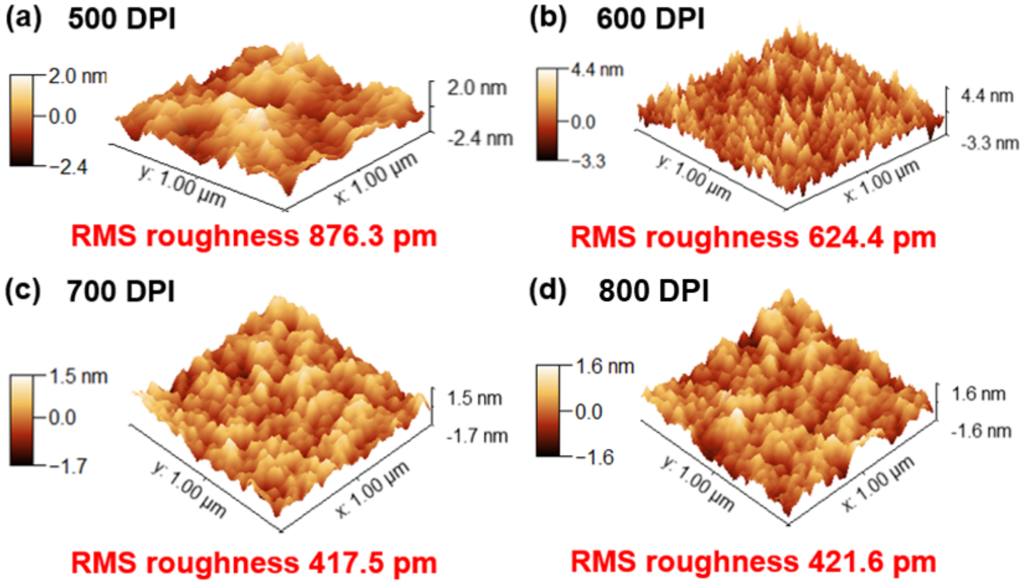

Supplementary Figure S8. AFM height images of a IJP thin film. a 500 DPI with R_q of 876.3 pm. **b** 600 DPI with R_q of 624.4 pm. **c** 700 DPI with R_q of 417.5 pm. **d** 800 DPI with R_q of 421.6 pm.

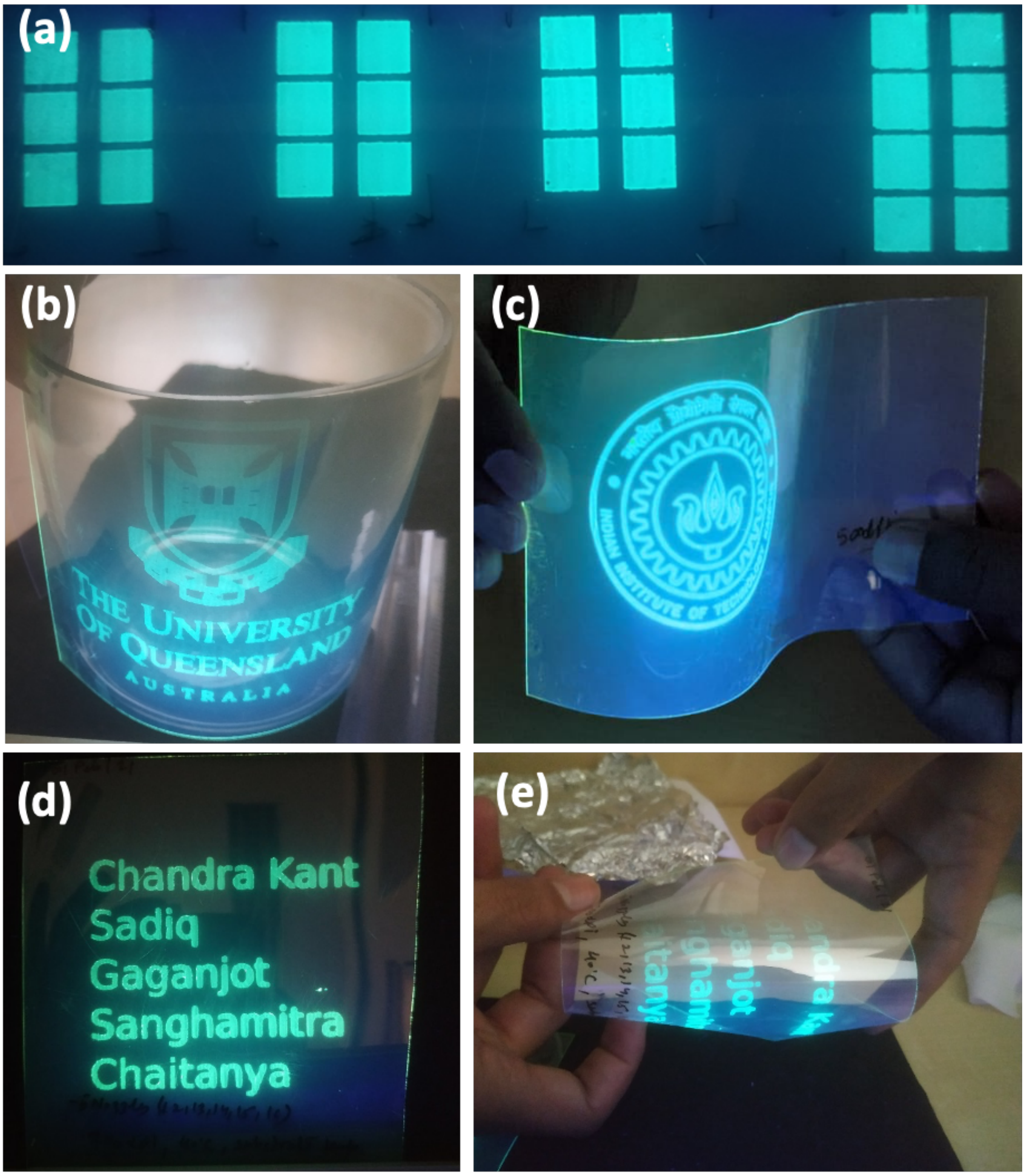

Supplementary Figure S9. Images of Inkjet-printed TADF ink on PET substrates a) pixel area 10 mm x10 mm (700 DPI), b-c) 80 mm x80 mm area (600 DPI) printed university logo in a flexible condition, d-e 50 mm x 50 mm area (700 DPI) texts. Images are illuminated with UV-lamp (365 nm).

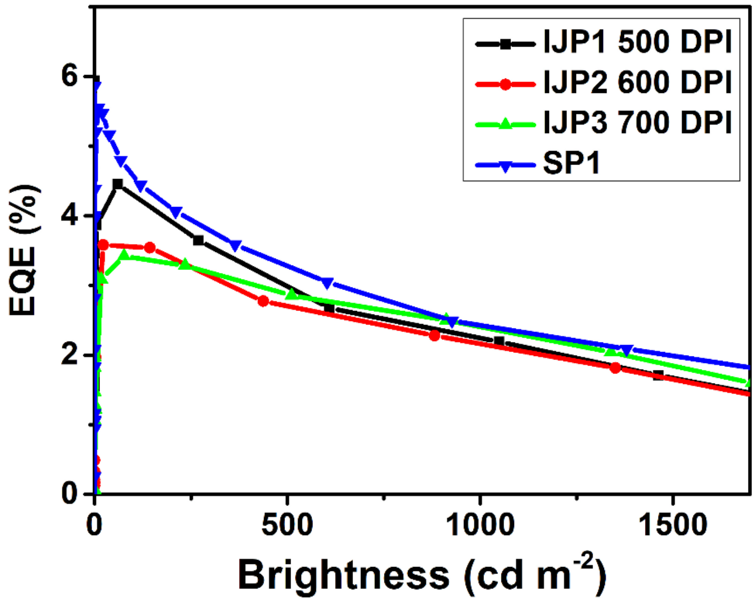

Supplementary Figure S10. External quantum efficiency *versus* luminance of the inkjet-printed OLEDs with DPI of 500, 600 and 700 for IJP1, 2 and 3, respectively) and spin-coated devices (SP1). The EQE of the devices was mesured from the front side of the device assuming Lambertian emission.

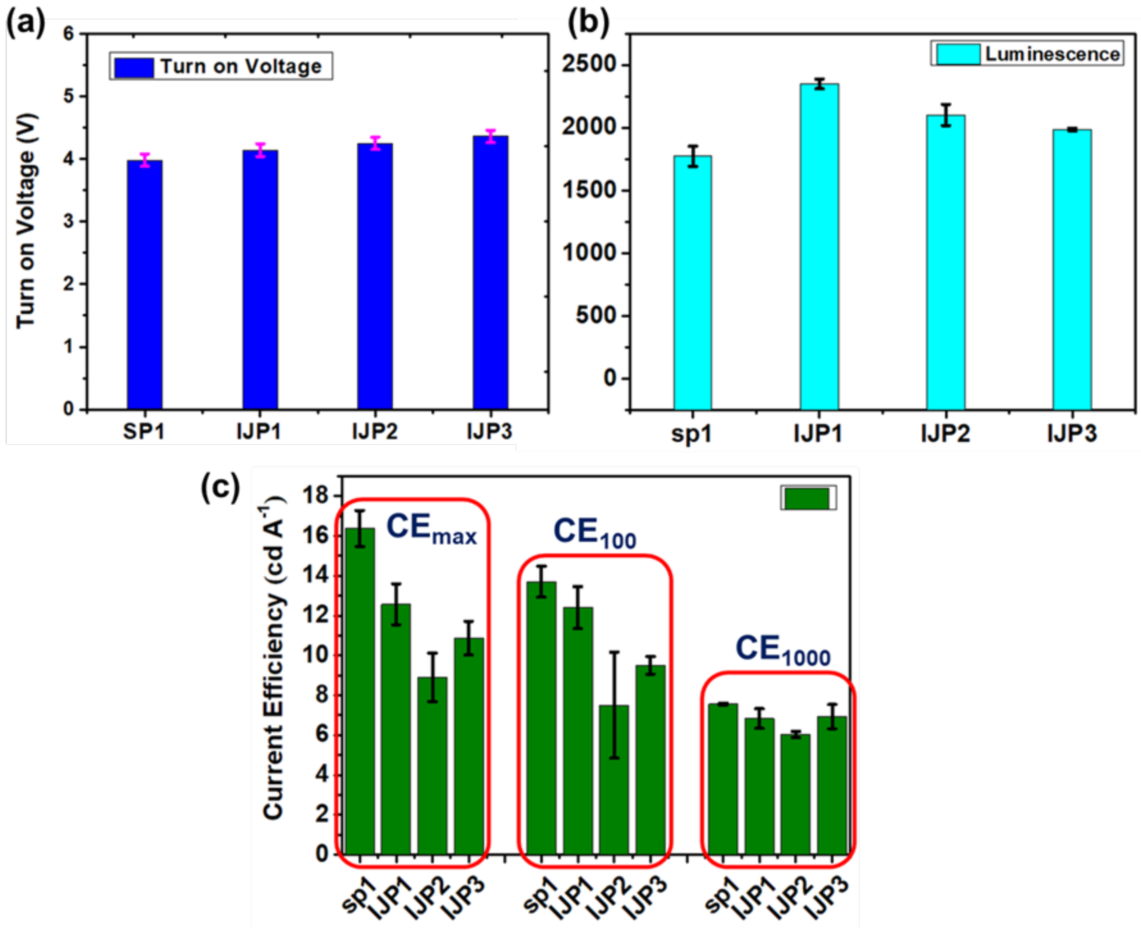

Supplementary Figure S11. Performance statistics of small area OLEDs comparing IJP and spin-coated (SP) devices. The device performance metrics were obtained from 3 to 4 pixels on one substrate. **a** Light turn-on voltage variation of spin coated and IJP devices measured at ~1 cd m^{-2}. **b** Brightness from spin coated and IJP OLEDs. **c** Current efficiency performance of IJP and spin coated OLEDs. The maximum current efficiency CE_{max} is quoted for a luminance of greater or equal to ~10 cd m^{-2}. CE_{100} and CE_{1000} is the current efficiency at 100 cd m^{-2} and 1000 cd m^{-2}, respectively.

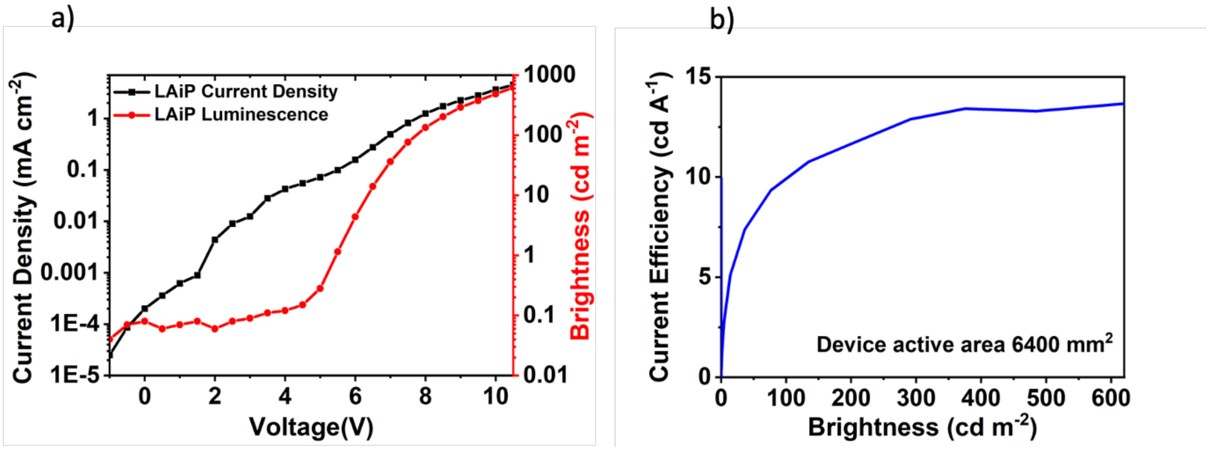

Supplementary Figure S12. Device characteristics of large area inkjet-printed panel (LAiP) OLEDs. a Current density and the brightness *verses* voltage of inkjet-printed large-area panel (LAiP) OLED with 80 × 80 mm^2 active area. **b)** Current efficiency *verses* brightness of the LAiP OLED.

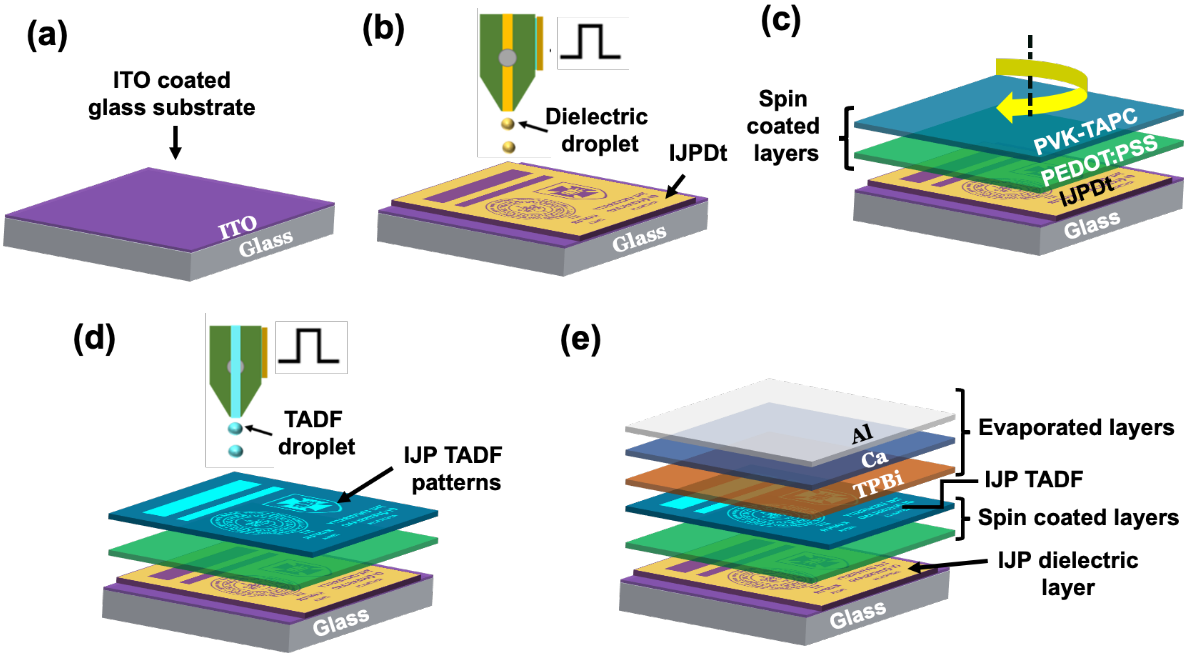

Supplementary Figure S13. Maskless IJP patterned large-area (100 × 100 mm^2) device fabrication steps. a pre-treated ITO-glass substrate. b inkjet printing of SU-8 (800 DPI, 1 µm thickness), a negative photoresist, to create the intricate template on the ITO substrate, followed by annealing at 80 °C for 5 min and then UV exposure to crosslink the SU-8. c spin coating of PEDOT:PSS and PVK-TAPC layer on ITO/SU-8 substrate. d IJP of TADF ink on top of the PVK-TAPC layer to create light emission regions. e thermal deposition of ETL TPBi, and Ca as well as an aluminium electrode.

Solvents	Boiling Point (°C)	Surface Tension (mN m^{-1})	Viscosity (cP)	Density (g cm^{-3})	Vapour Pressure (MPa)
DCE	83.6	38.7	0.82	1.24	10.24
CB	132.0	33.0	0.80	1.11	1.46
o-DCB	180.0	36.6	1.32	1.30	0.16
Toluene	110.6	28.0	0.81	0.86	3.80
MB	199.6	37.6	2.07	1.08	0.38

Supplementary Table S1. Boiling point, surface tension, viscosity, density, and vapour pressure of the solvents at room temperature (25 °C)[2,3].

Ink Conc. (mg mL^{-1})	Surface Tension (mN m^{-1})	Ink Viscosity (cp)	Observations
5.5	34	1.6	Stable for more than a month at room temperature
11.2	33	1.6	Nicely dissolved at room temperature
15.0	32	1.7	Dissolved at 40 °C but settled after a day
20.0	33	1.8	Ink settled; dissolved at 60 °C but settled again at room temperature

Supplementary Table S2. Ink properties and stability observations with differing concentrations of TADF/CBP in toluene:MB (40:60)

Ink composition	Viscosity (cP)	Surface Tension (mN m^{-1})	Density (g cm^{-3})	Z number
Toluene/MB (40:60)	1.8	33	0.998	12.9

Supplementary Table S3. Viscosity, surface tension, density & Z number of the ink studied.

Supplimetary References:

1. Material, L. E., Emitter, F. & Device, L. E. (12) United States Patent R3 O X O R6. **2**, (2017).
2. De Lanty, P. Solubility parameters. *OCL - Ol. Corps Gras Lipides* **12**, 299–301 (2005).
3. Kim, H. J., Lee, E. Y., Kim, M. Y., Kim, J. S. & Chin, B. D. Ink formulation and optimum surface condition for a printed organic light-emitting diode with high boiling point and surface tension solvents. *ECS J. Solid State Sci. Technol.* **10**, 106005 (2021).
4. Tao, R. *et al.* Homogeneous surface profiles of inkjet-printed silver nanoparticle films by regulating their drying microenvironment. *J. Phys. Chem. C* **121**, 8992–8998 (2017).
5. Brutin, D. & Starov, V. Recent advances in droplet wetting and evaporation. *Chem. Soc. Rev.* **47**, 558–585 (2018).
